# The Role of Altered Mitochondrial Metabolism in Thyroid Cancer Development and Mitochondria-Targeted Thyroid Cancer Treatment

**DOI:** 10.3390/ijms23010460

**Published:** 2021-12-31

**Authors:** Siarhei A. Dabravolski, Nikita G. Nikiforov, Alexander D. Zhuravlev, Nikolay A. Orekhov, Liudmila M. Mikhaleva, Alexander N. Orekhov

**Affiliations:** 1 Department of Clinical Diagnostics, Vitebsk State Academy of Veterinary Medicine [UO VGAVM], 7/11 Dovatora Street, 210026 Vitebsk, Belarus; 2 AP Avtsyn Research Institute of Human Morphology, 3 Tsyurupa Street, 117418 Moscow, Russia; nikiforov.mipt@googlemail.com (N.G.N.); zhuravel17@yandex.ru (A.D.Z.); mikhalevalm@yandex.ru (L.M.M.); 3 Center for Precision Genome Editing and Genetic Technologies for Biomedicine, Institute of Gene Biology, Russian Academy of Sciences, 34/5 Vavilova Street, 119334 Moscow, Russia; 4 Laboratory of Angiopathology, Institute of General Pathology and Pathophysiology, Russian Academy of Medical Sciences, 125315 Moscow, Russia; 5 Institute for Atherosclerosis Research, Osennyaya Street 4-1-207, 121609 Moscow, Russia; fuper@gmail.com (N.A.O.); a.h.opexob@gmail.com (A.N.O.)

**Keywords:** thyroid cancer, mitophagy, mitochondrial DNA mutation, mitochondrial dynamics, apoptosis

## Abstract

Thyroid cancer (TC) is the most common type of endocrine malignancy. Tumour formation, progression, and metastasis greatly depend on the efficacy of mitochondria—primarily, the regulation of mitochondria-mediated apoptosis, Ca^2+^ homeostasis, dynamics, energy production, and associated reactive oxygen species generation. Recent studies have successfully confirmed the mitochondrial aetiology of thyroid carcinogenesis. In this review, we focus on the recent progress in understanding the molecular mechanisms of thyroid cancer relating to altered mitochondrial metabolism. We also discuss the repurposing of known drugs and the induction of mitochondria-mediated apoptosis as a new trend in the development of anti-TC therapy.

## 1. Introduction

Thyroid cancer (TC) is one of the most common types of endocrine malignancy worldwide with steadily growing incidence and mortality rates during the last decades. An increase in the number of occurred incidences could be explained by the improved diagnostic techniques (allow to detect small-size tumours) but also because of the more often detection of large-size tumours via palpation [1]. TC develops from two cell types in the thyroid gland: >90% is derived from the follicular cells–epithelial cells, which are responsible for iodine uptake and thyroid hormone biosynthesis; 3–5% is derived from parafollicular cells, which are responsible for the biosynthesis and secretion of hormone calcitonin (known as MTC (medullary thyroid carcinomas)) [2,3]. Furthermore, based on the cellular morphology and histological architecture, thyroid follicular cells-derived carcinomas could be divided into four main types: carcinomas that retain differentiated properties are defined as DTC (differentiated thyroid carcinomas) and ATC (anaplastic thyroid carcinoma). Subsequently, DTCs are subdivided into well-differentiated subtypes (PTC (papillary thyroid carcinoma) and FTC (follicular thyroid carcinoma)) and the rarest and aggressive subtypes—PDTC (poorly differentiated thyroid carcinoma) [4]. The current view suggests that ATC is a result of mutations accumulation in ongoing WDTC, with PDTC as an intermediate state. However, some studies support also the idea that ATC could emerge de novo [5].

PTC, as the most common type of differentiated thyroid carcinoma (up to 80–85% cases), could be further classified into several variants: CV (classical variant), FV (follicular variant), and TCV (tall cell variant). PTC has mostly been associated with mutations in one of the MAPK (mitogen-activated protein kinase) signalling pathways: ALK (anaplastic lymphoma kinase), NTRK (neurotrophic receptor tyrosine kinase), and RET (rearranged during transfection) tyrosine kinase receptor, or RAS (rat sarcoma) and BRAF (rapidly accelerated fibrosarcoma type-B). The current data suggest that one mutation is sufficient to promote transformation [6]. FTC is a less common subtype of WDTC with 10–15% cases of all the thyroid carcinomas, driven by RAS, BRAF^V600E^ mutations, and PAX8/PPARγ (Paired Box Gene 8/Peroxisome Proliferator-Activated Receptor Gamma) rearrangements. Both PTC and FTC have a good prognosis with an overall 5-year survival of 90% and higher, although cases of tumours recurrence, spreading, and metastases are known and present a bad prognosis [7].

Mostly, WDTCs have a good prognosis and are treated by surgical resection in combination with radiotherapy, chemotherapy, and targeted drugs (although metastasis and recurrence could occur in 10–30% of PTC cases). However, all types of treatments are often associated with different negative side effects [8]. Since BRAF^V600E^ is the most prevalent mutation driving PTC initiation and progression, it could be treated with an FDA-approved BRAF^V600E^ inhibitor, vemurafenib [9]. Metastatic, radioactive-iodine-refractory WDTC, and advanced MTC could be treated with TKIs (tyrosine kinase inhibitors) (currently, there are four that are FDA-approved). However, treatment with TKIs is extremely expensive and associated with significant side effects [10].

PDTC and ATC are very rare and aggressive types of thyroid carcinomas with low survival rates (1–7%) and no available treatment options [11]. Mutations of both MAPK and PI3K (Phosphoinositide 3-Kinase Alpha) signalling pathways could be found in these types of tumours, along with mutations in other genes, which are associated with aggressiveness (such as *TP53* (Tumour Protein P53) and *EIF1AX* (Eukaryotic Translation Initiation Factor 1A Pseudogene 1) genes), mutations, and/or epigenetic modifications in the *TERT* (Telomerase Reverse Transcriptase) gene promoter. In addition, alteration in the expression of genes responsible for iodine transport and metabolism (such as *NIS* (Sodium/Iodide Symporter) or *SLC5A5* (Solute Carrier Family 5 Member 5)) are frequent, so the tumours could not accumulate iodine and respond to radioactive iodine therapy, which resulted in negative prognosis [12].

Tumorigenesis is a complex process involving the activation of oncogenes, inactivation of tumour suppressors, and reprogramming of cell death. Mitochondria play a crucial role in tumorigenesis via energy production, ROS (reactive oxygen species) generation, regulation of apoptosis, and Ca^2+^ metabolism. In addition, mitochondria quality control through mitophagy is associated with tumours’ growth and proliferation [13].

In this review, we focus on the recent progress in understanding the role of mitochondria in the molecular mechanisms of thyroid cancer development and progression. In addition, promising anti-TC medications (re-purposed existing drugs, natural compounds, and TC-specific) with defined molecular effects on mitochondria will be discussed.

## 2. The Role of Mitochondria in Tumorigenesis

Cancer cells have an energy metabolism different from that of normal cells, which could be explained by the high energy demand of progressing tumour cells. Intensive investigation of the molecular carcinogenesis of thyroid cancer during the last decades suggests that oncogenes and other tumour-related factors are targeted mainly on the regulation of cellular energy metabolism [14]. In addition to the production of 90% of the cellular energy, mitochondria also participate in Ca^2+^ metabolism, the regulation of cellular proliferation and apoptosis, lipid metabolism and urea cycle, synthesis of porphyrin and steroid hormone, and amino acids interconversion [15,16]. Further in this section, we discuss functional and structural aspects of mitochondrial dysfunction in thyroid tumorigenesis and tumour progression. The role of mutations in nuclear-encoded genes in thyroid cancer progression has been extensively studied [17,18]. In addition, the role of mitochondrial DNA mutations and alterations has been explored mostly on the rare and aggressive type of thyroid cancer—Hürthle cell carcinoma (or oxyphilic cell carcinoma) [19,20]. Thus, those topics will be excluded from the current review.

### 2.1. Mitochondrial DNA Alterations in TC

In contrast to the nuclear gene mutations, the role of mitochondrial DNA alteration in the development of thyroid cancer has not been well studied. Mitochondrial DNA encodes only 37 genes: 22 tRNAs (transfer RNA), 13 components of the electron transport chain, and 2 ribosomal RNAs. Some MtDNA mutations could influence mitochondria metabolism and biogenesis, and, potentially, lead to oncocytic phenotype [21]. It is known that thyroid tumours contain a very high number of mitochondria, and mutations in the mitochondrial complex I of the respiratory chain are most common in parathyroid cancer. Several recent publications support this idea. A total of 33 pathogenic mutations were identified among Chinese PTC patients and were associated with older age and advanced tumour stage. In particular, PTC occurrence was associated with eight SNP (single-nucleotide polymorphisms) sites: Mt16164(A > G), Mt16266(C > T), Mt5460 (G > A), Mt6680 (T > C), Mt9123 (G > A), Mt14587 (A > G), Mt16362 (T > C), and Mt709(G > A) [22]. Similarly, 25 mtDNA mutations were found in Chinese TC patients, and 60% of these mtDNA mutations were in genes encoding respiratory complex subunits [23]. A recent study on Austrian TCV patients suggests a strong statistical association between BRAF^V600E^ mutation and both pathogenic mtDNA mutations and loss of complex I integrity [24].

In addition to mtDNA mutations, tumour cells’ mtDNA-CN (mtDNA copy number) was associated with somatic mutations and clinical features in Chinese parathyroid carcinoma and adenomas patients [25]. Similarly, leukocyte mtDNA-CN was associated with an increased level of 8-OhdG (8-Oxo-2′-deoxyguanosine), which is a biomarker for oxidative DNA damage, and PTC and FTC types of thyroid cancer in the Chinese population. Thus, leukocyte mtDNA-CN could correlate with oxidative DNA damage and be used as an independent risk factor for TC [26]. These results were confirmed in another study, where the low content of circulating cell-free mtDNA was associated with PTC among Polish patients [27].

Other research suggests that some mtDNA haplogroups have a protective role for thyroid cancer. The investigation of mtDNA haplogroups in a population from south-eastern Europe suggests that haplogroup K and two mtDNA variants (Mt16224 (T > C) and Mt16261 (C > T)) were associated with a protective role for thyroid cancer [28].

Moreover, the content of mitochondria-associated proteins could be used as a biomarker for TC. For example, TCV has increased the expression of prohibitin, which is a protein required for optimal mitochondrial morphology and function [24]. Different TCs were characterised by an abnormally high abundance and chemical diversity of cardiolipins, which is a crucial component of the inner mitochondrial membrane, where it is involved in mitochondrial energy metabolism and required for the optimal function of numerous enzymes [29].

In total, a strong association was found between mtDNA mutations and TC. These mutations altogether with an examination of mtDNA content (cell-free circulation or in leukocyte), mtDNA haplogroup, and mitochondrial proteins (such as prohibitin and diversity of cardiolipins) could be used as an additional biomarker to prove TC and decrease the number of unnecessary thyroid manipulations.

### 2.2. Mitochondrial Quality Control and Mitophagy in TC

The biogenesis of new mitochondria is balanced with mitophagy, a specialised form of autophagy, which is regulating mitochondrial mass, quality, and proper functioning via the selective degradation of malfunctional and/or damaged mitochondria. There are two main types of mitophagy: receptor-mediated (orchestrated by BNIP3 (BCL2 Interacting Protein 3) and NIX (NIP-3-Like Protein X), FUNDC1 (FUN14 Domain Containing 1), BCL2L13 (BCL2 Like 13) and others), and Ubiquitin-mediated (PINK1 (PTEN-Induced Kinase 1) and Parkin (Parkin E3 Ubiquitin Protein Ligase)) reviewed in [30]. It was shown that PTC cells have a higher expression of genes associated with energy and mitochondrial-related pathways, autophagy, and mitochondrial fusion. On the contrary, the expression of mitophagy-related genes was decreased [31]. Mitochondrial dynamics involve coordinated cycles of fission and fusion, which are maintaining their shape, distribution, and size. The molecular processes of mitochondrial dynamics are governed by MFN2 (mitofusin 2), OPA1 (optic atrophy 1), FIS1 (mitochondrial fission 1), and DRP1 (dynamin-related protein 1), which are overexpressed in tumour cells and crucial for the development of the malignant phenotype. Accordingly, a blockade of Drp1 leads to decreased oncocytic cell migration and invasion [32].

Recently, another non-canonical p53-inducible mitochondrial quality control system was discovered. Mieap (mitochondria-eating protein, *SPATA18* gene), a p53-inducible protein, induces the accumulation of lysosomal proteins within damaged mitochondria to dispose of oxidised mitochondrial proteins to repair damaged mitochondria. Furthermore, Mieap induces vacuole-like structures to degrade too damaged mitochondria. Interestingly, the Mieap mechanism is not mediated by canonical autophagy proteins (reviewed in [33]). TC cells have a defective expression of MIEAP, which causes an increase in the number of abnormal mitochondria, ROS levels, mtDNA/nuclear DNA ratios, and acidification of the cytoplasm. Apparently, in TC, impaired mitochondrial function promotes a compensatory increase in de novo mitochondria biogenesis. On the other side, abnormal mitochondria could not be efficiently removed by both defective mitophagy systems (canonical and MIEAP-mediated), thus also contributing to the tumour growth and progression [34].

### 2.3. Mitochondrial Biogenesis and Metabolism in TC

Mitochondrial biogenesis is a complex and multistep cellular process regulated by NO (nitric oxide) and Ca^2+^ via PGC-1-related co-activators (PPARG Coactivator 1 Alpha) in human FTC [35]. Carcinogenesis in TC is associated with the abnormal proliferation of mitochondria, which could further affect different tissues.

Recent research suggests that in PTC, elevated mitochondrial mass is combined with low complex I and high complex II–V levels [36]. The low level of complex I could be explained by the higher number of pathologic mutations in mtDNA, while high levels of complexes II–V may represent some kind of compensatory effect to attenuate the complex I deficiency. In addition, it is possible that tumour cells with reduced efficacy or the complete absence of complex I might have some survival advantage in comparison to tumour cells with normal complex I [36].

Mutations and SNP variants in the nuclear-encoded genes also could affect mitochondrial metabolism and act as a TC risk factor. Recently, among FNMTC (familial non-medullary thyroid cancer) patients, the mutations in *MYO1F* (Myosin IF) gene (c.400 G > A, p.Gly134Ser) was identified. Myosins are motor proteins, use ATP energy to generate the force on actin filaments, and are known to participate in different intracellular movements; thus, they are involved in the development of many diseases [37]. This mutation was associated with the fragmented mitochondrial network, increased mitochondrial mass, higher ROS production, and low ATP/ADP ratio [38].

**Figure 1:**
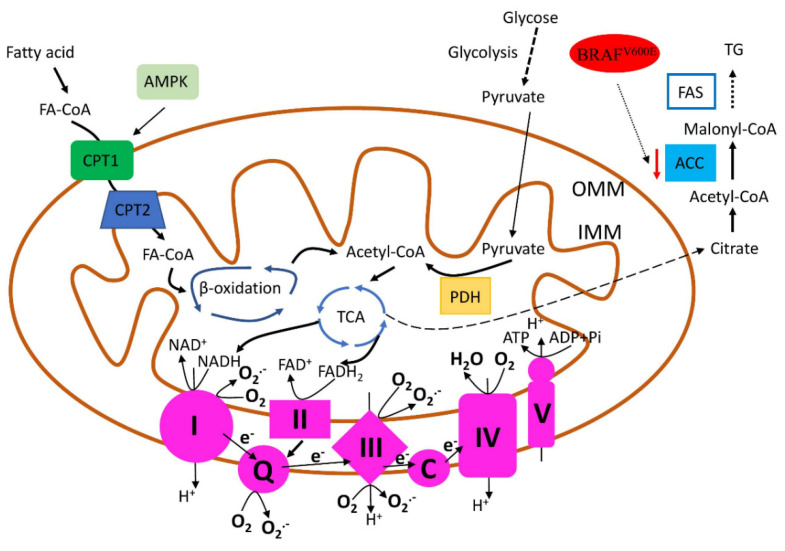
Mitochondrial FAO, ETC. Mutations in mtDNA lead to dysregulation in mitochondrial FAO, ETC, resulting in decreased ATP synthesis and increased ROS production. Effective energy generation (metabolised FA, supplied via FAO and TAC to ETC to produce ATP) is required for tumours’ development and progression. Regulation of the key FAO- (*CPT1* and *ACC1*) and ETC-related genes (*NOX4* and *COX4*) have been suggested as a promising strategy to decrease the efficacy of mitochondrial respiration of cancer cells. FAS—fatty acid synthesis; TG—triglyceride; PDH—pyruvate dehydrogenase; TCA—tricarboxylic acid cycle; I–V—components of mitochondrial ETC (respiratory) chain; Q—ubiquinone, coenzyme Q; C—cytochrome c.

Mitochondrial FAO (fatty acid oxidation) plays an important role in energy supply during tumours’ development and progression (Figure 1) [39]. Consequently, the higher expression of FAO-associated enzymes was identified in several types of tumours. CPT1 (carnitine palmitoyltransferase) is responsible for FA import into the mitochondria, and the high expression of CPT1 isoform Cpt1c is induced to promote cancer cell survival in conditions of metabolic stress [40]. PTC tissues are associated with a high level of Cpt1c expression, and Cpt1c up-regulation promotes cancer cell growth and metastasis. Cpt1 expression is regulated by AMPK (5′AMP-Activated Protein Kinase) activity and could be induced by metabolic stresses (such as low glucose and hypoxia). Since Cpt1c can protect cancer cells from stress-mediated death and contribute to PTC development and progression, the application of Cpt1c inhibitors (alone and in combination with AMPK agonists) could be used as a new promising way in PTC treatment [41].

Recent research has demonstrated that BRAF^V600E^ mutation is involved in lipid metabolism regulation via association with ACC2 (Figure 1) [42]. ACC (Acetyl-CoA Carboxylase) is the rate-limiting enzyme, catalysing the carboxylation of acetyl-CoA, which produces malonyl-CoA—the first reaction in de novo FAS (fatty acid synthesis). In addition, malonyl-CoA is an allosteric inhibitor of CPT1; therefore, it regulates FAO [43]. PTC cells carrying the BRAF^V600E^ mutation have down-regulated ACC2 expression, while the application of BRAF^V600E^ inhibitor vemurafenib increased ACC2 mRNA levels, de novo lipid synthesis rates, and decreased FAO in PTC-derived cells. Unfortunately, resistance to BRAF^V600E^ inhibitor vemurafenib is widely reported in patients [44]; therefore, ACC2 rescue may be used as a promising molecular strategy to overcome resistance to vemurafenib in PTC [42].

The growth and progression of tumour cells required O_2_, which could be supplied only by blood vessels. Limited O_2_ availability causes hypoxic conditions, which induce a higher expression of HIFs (hypoxia-inducible transcription factor). Mitochondrial ROS is a crucial factor in the stabilisation of HIF family members, which is associated with a metabolic shift from oxidative metabolism to glycolysis. NOXs (NADPH Oxidase) family members are functioning as the catalytic subunit of the NADPH oxidase complex and catalyse the reduction of molecular oxygen to various ROS. NOX4 is expressed at a high level in human TC cells and is regulated by TSH (thyroid-stimulating hormone) [45]. The heterodimerisation of NOX4 with the p22phox can increase ROS production [46]. A recent report suggests that NOX4 and p22phox in PTC are necessary for mitochondrial ROS production during hypoxia. NOX4 also affect other mitochondrial functions, such as oxygen consumption and membrane potential, thus acting as a glycolytic regulator of the PTC metabolism and proliferation [47].

COX (cytochrome c oxidase) is the terminal complex of the mitochondrial ETC (electron transport chain); it catalyses the transfer of electrons from cytochrome c to molecular oxygen. COX4 is the largest nuclear-encoded subunit, which is known to inhibit the enzyme activity at high ATP concentrations [48]. Recent research has demonstrated that metastatic MTC cells have an increased expression of COX4, which is correlated with tumour size and lymph node metastases. Interestingly, COX4 silencing in MTC-derived cells was associated with an inhibited MAPK pathway, decreased oxygen consumption, and ATP production, while having no effects on WDTC cells. Similarly, potassium cyanide treatment inhibited mitochondrial respiration and induced apoptosis in MTC-derived cells, but it had a minimal effect on WDTC cells. Since metastatic MTC cells are more sensitive to COX4 silencing, it could suggest COX4-targeted therapy as a promising tool against MTC [49].

To sum up, TC cells have a high demand for energy, which is achieved with a deep reprogramming of mitochondrial metabolic pathways, primarily ETC and FAO. The key enzymes in these pathways (COX4, NOX4 and CPT1, ACC2, respectively) (Figure 1) could be used as a promising target to regulate mitochondrial functions and slow down the proliferation of TC cells.

### 2.4. Mitochondria-Dependent Apoptosis in TC

**Figure 2:**
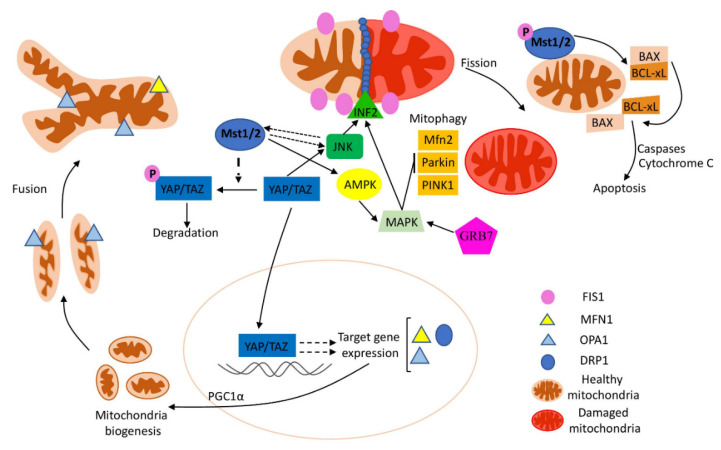
The role of the Hippo pathway in the regulation of mitochondrial biogenesis, mitochondria-mediated apoptosis, mitochondrial dynamics, and mitophagy. When the Hippo pathway is ‘‘off’’, YAP combines with TAZ to target the expression of mitochondria-related genes (to regulate mitochondrial biogenesis, dynamics, and mitophagy). In addition, the components of the Hippo pathway closely interact with the JNK/MAPK pathway to regulate mitochondrial dynamics and mitophagy. Arrows or blunt ends indicate regulation or inhibition, respectively, dashed arrow—skipped steps/components in the pathway. Drp1—dynamin-related protein 1, Mfn1/2—mitofusin1/2, OPA1—optic atrophy protein 1, INF2—inverted formin 2, JNK—c-Jun N-terminal kinase, MAPK—mitogen-activated protein kinase, PGC1α—peroxisome proliferator-activated receptor-a coactivator 1, PINK1—PTEN-Induced Kinase 1, YAP—Yes-Associated Protein 1, TAZ—Tafazzin, BAX—BCL2-Associated X, BCL-xL—Apoptosis Regulator Bcl-X, GRB7—Growth Factor Receptor Bound Protein 7.

Apoptosis initiation could be intrinsic (mitochondria-mediated) or extrinsic, which is activated by extracellular ligands binding to cell-surface death receptors. Both pathways required the involvement and activation of specialised proteases–caspases, which degrade proteins and kill the cell (reviewed in [50]). Additionally, a caspase-independent apoptosis mechanism was described. In this mechanism, mitochondria-localised AIF (apoptosis-inducing factor) protein released from damaged mitochondria moved to the nucleus and triggered chromosomes’ condensation and DNA fragmentation, thus initiating cell death [51]. The hippo signalling pathway, which is involved in the regulation of cell proliferation and apoptosis, could modulate TC cells’ viability and mitochondrial functions (Figure 2) [52]. Experiments on components of the Hippo pathway (combined overexpression of Mst1 (Macrophage Stimulating 1) and knock-down of Yap (Yes-Associated Protein 1)) resulted in the suppression of cancer cell migration and proliferation, activation of caspase-9-related apoptosis, respiratory function, ATP production, and mitochondrial membrane potential. In addition, mitochondrial fission was improved in the JNK–MIEF1 (c-Jun N-terminal kinases/Mitochondrial Elongation Factor 1)-dependent pathway [53].

Mst2 (Macrophage Stimulating 2), another Hippo pathway component, is a serine protease that is responsible for post-transcriptional phosphorylation, the regulation of mitochondrial ROS production, and the differentiation of the epididymal initial segment (via the MAPK pathway) [54,55]. Mst2 overexpression in TC cells leads to JNK pathway-mediated ER stress, which is associated with the subsequent rise of caspase-12 activity, increased mitochondrial oxidative stress, higher apoptotic rate, and reduced cell viability. These data suggest that Mst2 is a novel tumour-suppressor protein, which is acting via the JNK pathway and causes ER stress and mitochondrial damage [56].

Tafazzin is a co-activator of the Hippo pathways, which is responsible for the transcription of multiple tumorigenesis genes (such as *cyclin D*1 and *CCN2* (connective tissue growth factor)); thus, they are involved in cancer development and progression [57]. In addition, Tafazzin is involved in the regulation of many mitochondrial functions: respiratory activity (via sustaining the levels of cardiolipin), mitochondrial oxidative stress, and fission (reviewed in [58]). Up-regulated Tafazzin acts together with YAP to promote the EMT (epithelial–mesenchymal transition), cell cycle transition, and immunoresistance, thus promoting tumour growth, survival, and metastasis [52]. INF2 (inverted formin 2), a recently identified mediator of mitochondrial fission, is activated by the AMPK and/or JNK signalling pathway. Furthermore, INF2 promotes F-actin assembly into a constrictions ring to divide mitochondria into several smaller fragments [59]. Recent research shows that Tafazzin deletion leads to apoptosis-mediated death in TC cells [60]. Investigation of the exact molecular mechanisms suggests that Tafazzin deletion resulted in a dysregulation of mitochondrial energy metabolism, rise in ROS production, and initiated mitochondrial apoptosis. In addition, Tafazzin activates INF2 via the JNK signalling pathway (Figure 2). Respectively, the blockade of JNK prevented Tafazzin-mediated INF2 activation and increased the survival rate of cancer cells [60].

Recently, the role of GRB7 (Growth Factor Receptor-Bound Protein 7) in the regulation of cell cycle, proliferation, and mitochondrial apoptosis of TC cells via the MAPK pathway was shown [61]. GRB7 is an adaptor protein involved in protein–protein and protein–lipid interactions with different signalling molecules and receptor tyrosine kinases, which allow GRB7 to participate in various signalling pathways. In particular, high GRB7 expression was shown for many types of human cancers, where it correlates with tumours’ survival and aggressiveness [62]. It was found that GRB7 is up-regulated in TC, while its silencing leads to cell cycle arrest and inhibited proliferation in TC cells. Mechanically, GRB7 silencing impairs the activity and expression of mitochondrial respiratory complex, with a subsequent decrease in ATP output, glucose uptake, and lactose production in TC. In addition, a low level of GRB7 leads to increased levels of caspase 3 and other pro-apoptotic proteins, thus inducing mitochondrial apoptosis in the MAPK pathway-mediated way [61].

The primary cilium, a non-motile, microtubule-based sensory organelle, is responsible for the reception of external mechanical and chemical stimuli from the environment and their transduction into the cell. The primary cilia of thyroid follicular cells play a crucial role in maintaining cell polarity and are directly linked with its malignant transformation [63]. Recent experiments on mouse models with thyrocyte-specific loss of primary cilia (*Tg-Cre;Ift88^flox/flox^*) resulted in up-regulated apoptotic cell death in thyroid follicular cells. Similarly, the silencing of genes, responsible for ciliogenesis (*KIF3A* (Kinesin Family Member 3A) and *IFT88* (Intraflagellar Transport 88)) in human TC cells lines promote the oligomerization and overexpression of VDAC1 (Voltage-Dependent Anion Channel 1), which is a mitochondrial outer membrane component involved in cytochrome c release, thus stimulating mitochondria-dependent apoptosis [64].

These findings suggest that apoptosis regulation and initiation via the Hippo/Tafazzin–JNK–INF2 axis could be considered as a promising target in the development of new therapies against TC (Figure 2). Additionally, *GRB7* and ciliogenesis-related genes could be used as prognostic markers and therapeutic targets to stimulate apoptosis in TC.

## 3. Thyroid Cancer Treatment

### 3.1. Mitochondrial Apoptosis Targeted Drugs

In recent decades, the number of diagnosed TC cases has rapidly increased globally. PTC, the most common subtype of TC, accounts for 80–90% of all TC cases and has the best survival prognosis [65]. However, while commonly used surgical resection, with or without radiotherapy and TSH suppression shows a good prognosis, those approaches have many postoperative complications and side effects of low TSH. For ATC, the most aggressive form of TC, the prognosis is very poor, and treatments options are very limited. Therefore, new candidates for TC treatment with high and specific antitumor activities are urgently required [66].

Currently, TKI (tyrosine kinase inhibitors) are the most used type of drug used to fight TC, several TKIs are FDA-approved to treat different TC (advanced MTC, metastatic, radioactive-iodine-refractory WDTC. However, this type of medication has significant side effects and high price [67], often losing its efficacy due to the resistance of TC, as it was shown for vandetanib and cabozantinib [68]. The other approach implies repurposing existing FDA-approved drugs that were not originally designed and not tested as an anti-cancer treatment. For example, an in vitro evaluation of an HIV protease inhibitor nelfinavir has demonstrated effectiveness against all histological types of TC. On the molecular level, nelfinavir downregulates the MAPK signalling pathway, inhibits proliferation, and induces DNA damage in WDTC and ATC cells, inducing apoptosis in MTC cells [69]. Similarly, metformin, a widely used anti-diabetes drug, has been demonstrated to inhibit RPS6KB1 (Ribosomal protein S6 kinase beta-1) protein and oxidative phosphorylation, up-regulate AMPK, and down-regulate *GPD2* (mitochondrial glycerol-3- phosphate dehydrogenase), thus inhibiting TC growth in vitro and in vivo [70].

Mitotane, a steroidogenesis inhibitor known to inhibit key enzymes of the mitochondrial respiratory chain, induces ER stress and apoptosis in ACC (adrenocortical carcinoma) cells [71]. Similarly, mitotane was effective against TC cells, where mitotane treatment leads to DNA damage, caspase-3 cleavage, and overexpression of pro-apoptotic and ER-stress marker genes. In addition, the mitochondrial membrane lost its potential, and the production of ATP was decreased [72].

Niclosamide, a derivative of salicylamide, is an oral anthelminthic drug with nearly 50 years of history. Recently, it was recognised that niclosamide has wide and diverse activities that could be used to induce apoptosis, interfere with cancer-driving signalling cascades, and inhibit the metastasis of different tumour cell types [73]. In PTC and ATC cell lines, niclosamide was shown to inhibit cell proliferation and induce apoptosis. Mechanically, Bax and caspase-3 were activated, while mitochondrial membrane potential and Bcl-2 were inhibited, suggesting apoptosis induction via the mitochondria-mediated pathway. In addition, matrix metalloproteinases 2 and 9 were down-regulated, and tissue inhibitor of metalloproteinase 2 was up-regulated, thus providing an inhibitory effect on cancer cells metastasis [74].

Mortalin (Heat Shock 70kDa Protein 9) is a heat shock protein detected in different subcellular compartments and involved in cell proliferation, stress response, and maintenance of the mitochondria. In addition, mortalin is often overexpressed in different types of cancers, where it facilitates tumour cell proliferation and survival [75]. PTC, FTC, and ATC are also associated with up-regulated mortalin, and mortalin depletion can lead to growth arrest and cell death in cell cultures of those types of TC [76]. The application of Mito-CP, the disrupter of mitochondrial metabolism [77], resulted in a robust induction of apoptosis in PTC and ATC cell lines. Interestingly, Mito-CP-mediated apoptosis was partially rescued by mortalin overexpression [76].

Taken together, these results demonstrated that existing approved drugs (such as mitotane, Mito-CP, and niclosamide) could be repurposed and considered as a novel agent for the treatment of TC. Due to the resistance of TC to the TKI, further investigation of the currently used medications and the development of new drugs with TC-specific mitochondria-targeted activities is required.

#### Natural Substances with Pro-Apoptotic Properties

Capsaicin, an active component of chilli peppers, is the agonist of TRPV1 (transient receptor potential vanilloid type 1), whose anti-proliferative and pro-apoptotic properties were studied on several kinds of cancer cells [78], including also TRPV1-mediated anti-metastatic activity on PTC cell culture [79]. Mechanically, capsaicin activates TRPV1 and causes Ca^2+^ entrance into the cell, resulting in the disturbance of intracellular Ca^2+^ homeostasis [80]. Recent research has shown that capsaicin is effective also against the ATC type of TC, where capsaicin treatment caused severe Ca^2+^ overload in mitochondria, increase in ROS production, mitochondrial membrane depolarisation, and opening of mitochondrial permeability transition pores. Subsequently, cytochrome c was released into the cytosol, and caspase activation led to apoptosis [81].

Berberine, a natural isoquinoline alkaloid, is found in many plants and exhibited multiple medical-relevant activities, including anti-carcinogenic [82,83]. PTC and ATC types of TC were more sensitive to berberine cytotoxic properties than normal thyroid cells with a more prominent inhibition of proliferation, induction of mitochondrial apoptosis, and cell cycle arrest. In TC cell lines, the levels of Bax/Bcl-2, P21, and cleaved caspase 3 were increased, while Cyclin E1, Vimentin, and CDK2 (Cyclin-Dependent Kinase 2) were decreased. In general, the expression of PI2K–AKT and MAPK pathways was decreased and disturbed, suggesting that berberine has a high potential for the treatment of TC [84].

Myricetin is a common flavonoid found in different berries and herbs. Previous studies have reported that myricetin has a wide range of biological properties: anti-oxidant, anti-viral, cytoprotective, anti-microbial, and anti-cancer [85]. In the case of PTC cells, myricetin was shown to induce mitochondrial dysfunction-mediated apoptosis. Myricetin treatment resulted in an up-regulation of caspase cascades proteins (caspases 3, 8, and 9), Bax/Bcl-2 ratio, alteration of the mitochondrial membrane potential, and AIF release [86]. A similar mechanism was described also for the ATC type of TC, where myricetin treatments caused DNA condensation, induction of mitochondria-mediated apoptosis, DNA condensation, and cell cycle arrest in the sub-G1 phase, with an overall reduction of cell proliferation by 70% [87].

In total, we could conclude that many existing medications and natural compounds could be used in TC treatment. The induction of mitochondria-mediated apoptosis via the modulation of mitochondrial Ca^2+^ levels is relatively well-studied and used in anti-cancer therapy [88]. Various natural compounds are known to have a wide range of biological activities and provide beneficial effects on many levels. With the proper investigation and understanding of the underlying molecular mechanisms, these properties could be useful in the development of anti-TC therapeutic agents in the future.

### 3.2. Mitochondrial Biogenesis and Metabolism Targeted Drugs

Growing evidence supports the significance of mitochondrial metabolism reprogramming for cancer cell proliferation and survival because they required more energy and building blocks for uncontrolled proliferation. However, such modifications and enhancements often lead to an increased production of toxins (primarily ROS) harmful to cancer cells. Such vulnerability provides a promising target to suppress cancer cells growth with no or minimal effect on normal healthy cells [89].

Tigecycline is an FDA-approved antibiotic binding to 30S ribosome subunit and is also known to suppress mitochondrial protein translation in mammalian cells, including also several types of cancer cells [90,91]. Tigecycline inhibited proliferation and induced apoptosis in PTC, ATC, and FTC cell lines and mice models without causing significant toxicity. In combination with the standard chemotherapeutic drug paclitaxel, tigecycline achieved better efficacy than paclitaxel alone in both in vitro and in vivo experiments. Mechanically, in TC cells, tigecycline inhibited mitochondrial respiration and ATP production more effectively than in normal thyroid cells, supporting the idea that TC cells require a higher level of mitochondrial biogenesis and ATP in comparison to normal thyroid cells [92].

Atovaquone is an FDA-approved antimalarial drug with a broad spectrum of anti-parasitic properties. It acts by inhibiting the cytochrome bc1 complex and mitochondrial respiration, leading to a blockage of energy supply. Recently, atovaquone was reported as a promising anti-cancer agent that is effective against cancer cells and cancer stem cells. The application of atovaquone increase radiosensitivity by alleviating tumour hypoxia, thus suggesting its high potential in combination with other anti-cancer drugs [93,94]. In ATC and FTC cell cultures, atovaquone decreases growth, migration, and survival by inhibiting mitochondrial complex III activity and subsequently reducing ATP production. In addition, atovaquone suppresses the phosphorylation of STAT3 (Signal Transducer and Activator Of Transcription 3) in TC cells, an important regulator of cell growth and apoptosis, which is a consequence of mitochondrial respiration inhibition [95]. Similarly, another anti-malarial drug Artesunate was shown to act against chemo-sensitive and -resistant ATC in vitro and in vivo. Artesunate inhibits growth and induces apoptosis, suppressing mitochondrial functions without affecting glycolysis in ATC cells [96].

Due to the high metabolic burden of actively growing TC, the key mitochondrial systems are overloaded. Several FDA-approved drugs have approved efficiency against TC via the inhibition and/or suppression of mitochondrial respiration, ATP production, and levels of released ROS. The application of these medications in combination with other anti-cancer drugs and therapeutical approaches is a new promising anti-TC strategy.

## 4. Conclusions

Mitochondrial abnormalities are playing a crucial role in the development and progression of all types of thyroid cancer. TC cell-specific mitochondria produce high levels of ROS under the in vivo hypoxic tumour microenvironment, which further causes oxidative damage to biomolecules, inducing genomic instability and metabolic reprogramming (fatty acid synthesis, oxidative phosphorylation, glycolysis, TCA cycle, and carbon metabolism). In addition, the mitochondrial ROS contribute to tumour growth, epithelial–mesenchymal transition, cancer invasion, and metastasis. Thus, further comprehensive and insightful investigations on TC-specific mitochondrial signalling pathways (such as regulating mitochondria-specific apoptosis and dynamics) would help to develop an effective method and/or strategy for the prevention, diagnosis, and therapy of different types of TCs.

## Abbreviation

ACC	acetyl-CoA carboxylase
ACC	adrenocortical carcinoma
AIF	apoptosis-inducing factor
ALK	anaplastic lymphoma kinase
AMPK	5′AMP-Activated Protein Kinase
ATC	anaplastic thyroid carcinoma
BCL2L13	BCL2 Like 13
BNIP3	BCL2 Interacting Protein 3
BRAF	rapidly accelerated fibrosarcoma type-B
CCN2	connective tissue growth factor
CDK2	Cyclin-Dependent Kinase 2
COX	cytochrome c oxidase
CPT1	carnitine palmitoyltransferase
CV	classical variant
DRP1	dynamin-related protein 1
EIF1AX	Eukaryotic Translation Initiation Factor 1A Pseudogene 1
FAO	fatty acid oxidation
FAS	fatty acid synthesis
FIS1	mitochondrial fission 1
FNMTC	familial non-medullary thyroid cancer
FTC	follicular thyroid carcinoma
FUNDC1	FUN14 Domain Containing 1
FV	follicular variant
GPD2	mitochondrial glycerol-3- phosphate dehydrogenase
GRB7	Growth Factor Receptor Bound Protein 7
HIFs	hypoxia-inducible transcription factor
IFT88	Intraflagellar Transport 88
JNK-MIEF1	c-Jun N-terminal kinases/Mitochondrial Elongation Factor 1
KIF3A	Kinesin Family Member 3A
MAPK	mitogen-activated protein kinase
MFN2	mitofusin 2
Mst1	Macrophage Stimulating 1
Mst2	Macrophage stimulating 2
MTC	medullary thyroid carcinomas
mtDNA-CN	mtDNA copy number
MYO1F	Myosin IF
NIS	Sodium/Iodide Symporter, or SLC5A5, Solute Carrier Family 5 Member 5
NIX	NIP-3-Like Protein X
NO	nitric oxide
NOXs	NADPH Oxidase
NTRK	neurotrophic receptor tyrosine kinase
OPA1	optic atrophy 1
Parkin	Parkin E3 Ubiquitin Protein Ligase
PAX8	Paired Box Gene 8
PDTC	poorly differentiated thyroid carcinoma
PGC-1α	PPARG Coactivator 1 Alpha
PI3K	Phosphoinositide 3-Kinase Alpha
PINK1	PTEN Induced Kinase 1
PPARγ	Peroxisome Proliferator-Activated Receptor Gamma
PTC	papillary thyroid carcinoma
RAS	rat sarcoma
RET	rearranged during transfection
RPS6KB1	Ribosomal protein S6 kinase beta-1
STAT3	Signal Transducer And Activator Of Transcription 3
TC	thyroid cancer
TCV	tall cell variant
TERT	Telomerase Reverse Transcriptase
TKI	tyrosine kinase inhibitors
TP53	Tumour Protein P53
TRPV1	transient receptor potential vanilloid type 1
TSH	thyroid-stimulating hormone
VDAC1	Voltage-Dependent Anion Channel 1
WDTC	well-differentiated thyroid carcinomas
Yap	Yes-Associated Protein 1
